# Dual-topology insertion of a dual-topology membrane protein

**DOI:** 10.1038/ncomms9099

**Published:** 2015-08-26

**Authors:** Nicholas B. Woodall, Ying Yin, James U. Bowie

**Affiliations:** 1 Department of Chemistry and Biochemistry, UCLA-DOE Institute, Molecular Biology Institute, University of California, Los Angeles, Los Angeles, California 90095-1570, USA

## Abstract

Some membrane transporters are dual-topology dimers in which the subunits have inverted transmembrane topology. How a cell manages to generate equal populations of two opposite topologies from the same polypeptide chain remains unclear. For the dual-topology transporter EmrE, the evidence to date remains consistent with two extreme models. A post-translational model posits that topology remains malleable after synthesis and becomes fixed once the dimer forms. A second, co-translational model, posits that the protein inserts in both topologies in equal proportions. Here we show that while there is at least some limited topological malleability, the co-translational model likely dominates under normal circumstances.

## Abstract (web summary)

Dual-topology membrane proteins consist of subunits that have identical sequence but reside in the membrane in two inverted orientations. Here, Woodall *et al.* find that the dual topology of the transporter EmrE is largely achieved by initial insertion in both topologies rather than major rearrangements after insertion.

## Graphical abstract

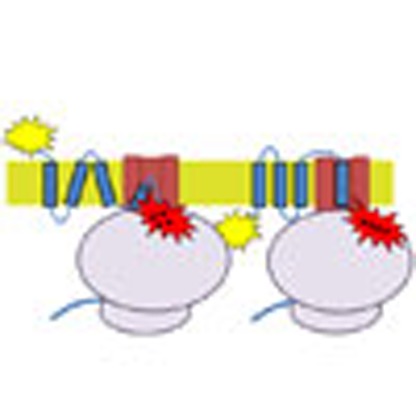

A dual-topology protein presents a difficult problem for evolution to solve. How can the same polypeptide achieve equal populations of the two topologies? If one orientation is strongly preferred it will lead to many orphan subunits that do not have an opposite topology partner. A possible solution to this problem would be for topology to remain malleable after synthesis so that the topology is set only after an opposite topology partner is found, driven by the stability of the antiparallel dimer^1^. We will refer to this as post-translational dual-topology generation (Fig. 1a). A number of studies have shown that membrane protein topology can be altered after synthesis. In particular, the Skach lab showed that aquaporin-1 can insert in a partially incorrect topology and then re-orient after synthesis^2^ and work from the Dowhan group revealed that altering the lipid composition leads to a remarkable topology rearrangement of an entire domain of lactose permease^3^,^4^.

The most well studied dual-topology transporter is the multidrug resistance protein EmrE^1^,^5^,^6^. In one subunit the N and C termini are inside the cytoplasm (N_in_/C_in_) and in the other subunit, the N and C termini are outside (N_out_/C_out_)^7^,^8^. The topology of helical membrane proteins can be predicted by the preference for positively charged amino acids to reside in the cytoplasm, the so-called positive-inside rule^9^,^10^. As a dual-topology protein, EmrE does not exhibit a positive-inside rule charge bias. By the strategic introduction of positive charges, however, it is possible to force subunits into either the N_in_/C_in_ topology or the N_out_/C_out_ topology^11^. Subunits forced into the N_in_/C_in_ topology will not form active dimers with themselves, but will form active dimers with subunits forced into the N_out_/C_out_ topology.

Studying the dual-topology protein EmrE, the von Heijne group showed that only subunits with N_out_/C_out_ topology would complement an EmrE construct with C-terminal positive charges^12^. These results have been interpreted to imply that topology can remain undefined until after the entire protein is made, consistent with the post-translational model (Fig. 1b)^12^,^13^,^14^. Yet these results can also be explained by a co-translational model in which the protein inserts in both topologies, but the C-terminal positive charges simply inactivate subunits initially inserted in the N_out_ topology (Fig. 1b). Consequently, without any direct examination of the inserted topologies, the indirect complementation experiments do not demonstrate topological malleability and the mechanism of dual-topology generation remains in question.

The problem with distinguishing between the post- and co-translational topology models is that the final state is the same: dual topology. What is needed is a way to lock the N terminus in its initially inserted topology. As described below, we find that placement of a short haemagglutinin (HA) epitope tag at the N terminus of the protein (HA-EmrE, Fig. 2a) allows the expression of both topologies of EmrE, but blocks subsequent topology rearrangement, allowing us to examine the co- and post-translational topology generation models. We find that some post-translational topology changes can occur, but that EmrE normally inserts in both N_in_ and N_out_ orientation with similar frequencies so that post-translational corrections are not a major mechanism for dual-topology insertion.

## Results

### HA tagging of EmrE does not prevent dual-topology insertion

We first asked whether the HA tag altered the functional behaviour of the protein by attempting to recapitulate the prior *in vivo* results from the von Heijne group which showed that C-terminal positive charges would only generate active N_in_/C_in_ subunits^12^. As shown in Supplementary Fig. 1a, HA-EmrE imparts resistance to ethidium bromide suggesting that HA-EmrE is functional and can therefore attain a dual topology. We then created a construct with a cluster of positive charges (KKKHHHHHH) at the C terminus, HA-EmrE-C+, to mirror the construct used previously^12^ (Fig. 2a) and a control construct, EmrE-C+, without the HA tag, leaving a wild-type N terminus. We then tested the ability of these constructs to complement subunits forced into either the N_in_/C_in_ topology (HA-EmrE_locked_in_) or the N_out_/C_out_ topology (HA-EmrE_locked_out_) by strategic placement of positive charges as described previously^11^. EmrE-C+ and HA-EmrE-C+ effectively complement the N_out_/C_out_ subunit HA-EmrE_locked_out_, but not HA-EmrE_locked_in_ (Fig. 2b). Thus, EmrE-C+ and HA-EmrE-C+ produce active subunits, but only in the N_in_/C_in_ topology, consistent with prior experiments^12^.

### Rapid degradation of orphan EmrE subunits

We next sought to move beyond genetic complementation to directly map the topology of the constructs. We were initially stymied in these efforts, however, because both HA-EmrE-C+ and EmrE-C+ had very low expression levels in the absence of the appropriate N_out_/C_out_ partner subunit. We therefore considered the possibility that orphan subunits might be rapidly proteolyzed. Figure 3a shows the degradation of HA-EmrE-C+ over time in the presence of the incorrectly oriented subunit partner, HA-EmrE_locked_in_, or in the presence of the correctly oriented subunit partner HA-EmrE_locked_out_. When dimer formation is precluded, the HA-EmrE-C+ is degraded rapidly (half-life <30 min), while in the presence of the appropriate partner, there is essentially no detectable degradation after 120 min. These results suggest that the C-terminally charged constructs exist in an unstable form that is subject to proteolysis.

### Blocking FtsH-mediated proteolysis of orphan EmrE subunits

FtsH is an integral membrane protease that is known to preferentially degrade unstable membrane proteins^15^, so we tested whether FtsH was involved in degrading HA-EmrE-C+. Figure 3b compares the expression levels in a control strain (AR3289) and an FtsH-null strain (AR3291)^16^. Expression is ∼80-fold higher in the FtsH-null strain than in the control strain, indicating that FtsH is at least partially responsible for the degradation of HA-EmrE-C+. The FtsH-null strain became an important tool for mapping the topological variants of EmrE, because it allowed us to map the topology of even unstable forms that might otherwise be lost to degradation.

With the ability to prevent proteolysis, we sought to directly determine the topology of EmrE-C+ constructs using cysteine accessibility. Cysteine accessibility maps the topology of a membrane protein by identifying whether a single introduced cysteine resides in the cytoplasm or the periplasm, by measuring the reactivity of the cysteine to a membrane-impermeable reagent^17^. The procedure is outlined in Supplementary Fig. 2. We first add the membrane-impermeable reagent, 4-acetamido-4′-maleimidylstilbene-2,2′-disulfonic acid (AMS), and later a membrane-permeable biotinylation reagent, 3-(*N*-maleimidopropionyl)-biocytin (MPB). If AMS has already reacted with the lone cysteine (that is, cysteine has a periplasmic location) then reaction of MPB will be blocked by AMS. Otherwise MPB will modify the cysteine. The extent of MPB modification can be detected by a gel shift in the presence of avidin (seen as a loss of the gel band corresponding to free EmrE). To reliably assess the level of biotinylation without AMS, we also perform a reaction with MPB alone. To apply this method, we introduced unique cysteines (3C, F27C, Q81C and T108C) into a cysless background (C39A/C41A/C95A), denoted as EmrE^Cless^. The EmrE^Cless^ variants were unreactive with MPB under our conditions and the intensity of the bands on western blots were linearly related to the amount of protein loaded (Supplementary Figs 3 and 4). We utilized the FtsH-null strain in all cysteine-accessibility topology experiments and three experimental replicates unless otherwise noted.

### The topology of EmrE-C+ is N_in_/C_in_

To assess the topology of EmrE^Cless^-C+ with a wild-type N terminus, we mapped the locations of F27C in the first loop and T108C on the C terminus. As expected for an N_in_/C_in_ topology, F27C is periplasmic as indicated by the large decrease in the avidin-dependent gel shift on prior reaction with AMS (78±13% change compared with no AMS, error is standard deviation observed for *n*=4 replicates) (Fig. 4a). The C-terminally located T108C showed no change in gel shift after reaction with AMS (10±14%, *n*=4) indicating a cytoplasmic location, as is expected for an N_in_/C_in_ topology (Fig. 4a). These results indicate that the C-terminal positive charges do define the topology of the N terminus as suggested previously based on complementation experiments^12^.

### Topology of HA-EmrE-C+

Continuing our characterization of the HA-tagged protein, we examined the topology of HA-EmrE^Cless^-C+ using the same F27C and T108C mutations. A cysteine introduced into the first loop (F27C) of HA-EmrE^Cless^-C+ showed only partial blocking (48±13%, *n*=5) by AMS (Fig. 4b). In contrast the C-terminal cysteine, T108C, which is adjacent to the C-terminal positive charges showed no response to AMS (1±4%, *n*=3), which indicates that it resides completely in the cytoplasm (Fig. 4b).

**Figure 1:**
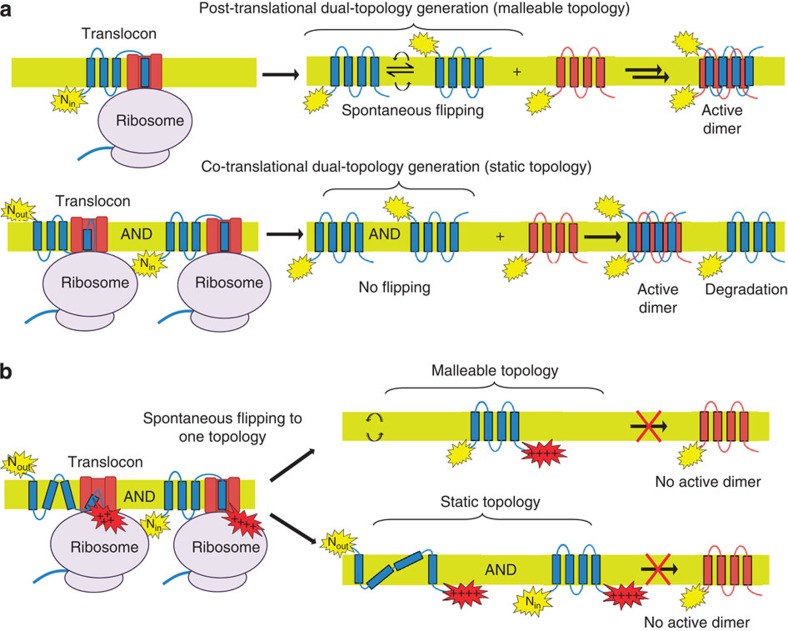
Dual-topology insertion models. (**a**) Post-translational dual-topology model: EmrE inserts into the membrane in a preferred topology and then changes topology to form the stable antiparallel dimer. Co-translational dual-topology model: EmrE obtains dual topology by inserting equally into both topologies. Extra subunits are degraded. (**b**) Topology of EmrE with C-terminal positive charges if the N-terminal helix has a malleable or static topology. Regardless of the mechanism, no active dimer can be formed with the N_in_/C_in_ EmrE. The frustrated N_out_/C_in_ topology shown is one of the many possible topologies.

**Figure 2:**
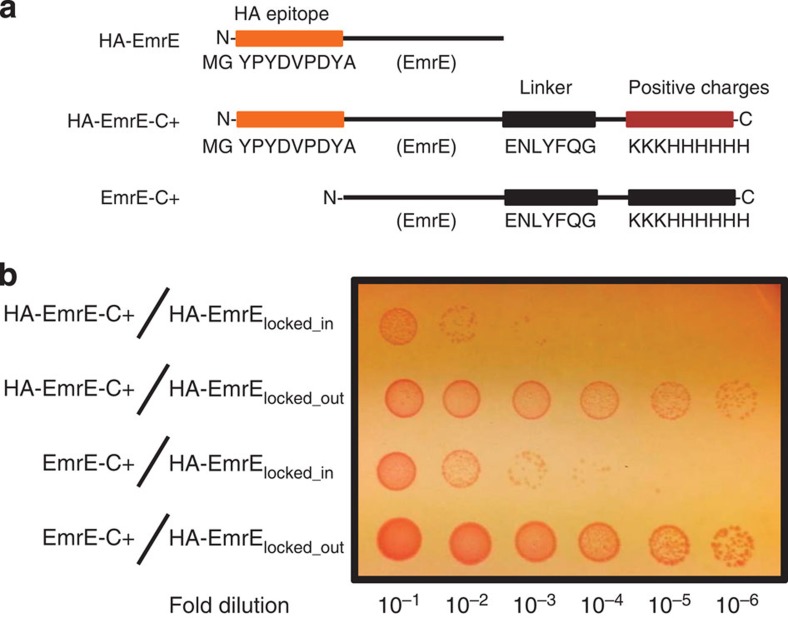
Indirect topology assay of EmrE-C+ and HA-EmrE-C+. (**a**) Diagram of the EmrE constructs used. (**b**) The growth of 10-fold dilutions of stationary phase cell culture expressing the HA-EmrE-C+ or EmrE-C+ constructs with a single topology EmrE mutant (HA-EmrE_locked_in_ or HA-EmrE_locked_out_), spotted on agar plates in the presence of 190 μg ml^−1^ ethidium bromide.

**Figure 3:**
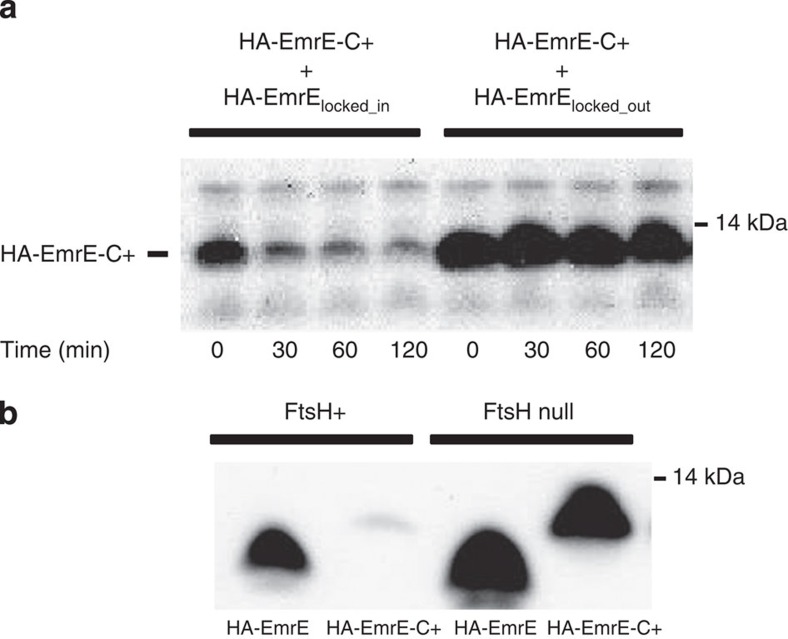
FtsH-dependent proteolysis of EmrE. (**a**) The loss of HA-EmrE-C+ co-expressed with either HA-EmrE_locked_in_ or HA-EmrE_locked_out_ after protein synthesis has been stopped at time zero. HA-EmrE –C+ was visualized by western blotting for the penta-histidine epitope. (**b**) The expression of HA-EmrE and HA-EmrE-C+ in wild type and FtsH knockout cells. The protein was visualized by western blotting using the HA epitope.

**Figure 4:**
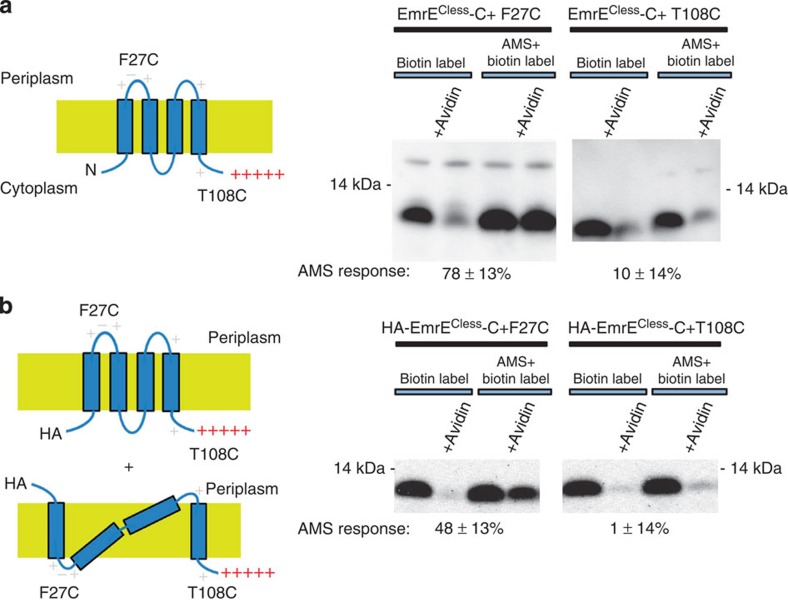
Direct-topology assay of EmrE-C+ and HA-EmrE-C+. Proposed topologies are shown on the left and cysteine-reactivity data are shown on the right. Added positively charged residues are shown in red. Invariant positively charged residues present in the wild-type protein are shown in light grey. AMS response is the per cent change in biotinylation due to the reaction with AMS. (**a**) Results for the EmrE^Cless^-C+ constructs. The high AMS response of F27C (*n*=4) is indicative of a periplasmic cysteine and the low AMS response of T108C(*n*=4) is indicative of a cytoplasmic location. (**b**) Results for the HA-EmrE-C+ construct. While T108C (*n*=3) shows a low AMS response similar to the EmrE^Cless^-C+ construct that does not possess and HA tag, F27C (*n*=5) shows much lower AMS response than seen in the EmrE^Cless^-C+ construct. EmrE^Cless^-C+ was visualized by western blotting for the penta-histidine epitope and HA-EmrE-C+ was visualized by western blotting for the HA epitope.

The partial reactivity of F27C in the HA construct led us to consider the possibility that the N terminus normally inserts in two different topologies, but that the HA tag blocks subsequent topology rearrangement, leading to a mixed topology form as illustrated in Fig. 1b. We first had to evaluate other possible reasons for the partial reactivity, however.

### Partial reactivity is not due to an environmental constraint

**Figure 5:**
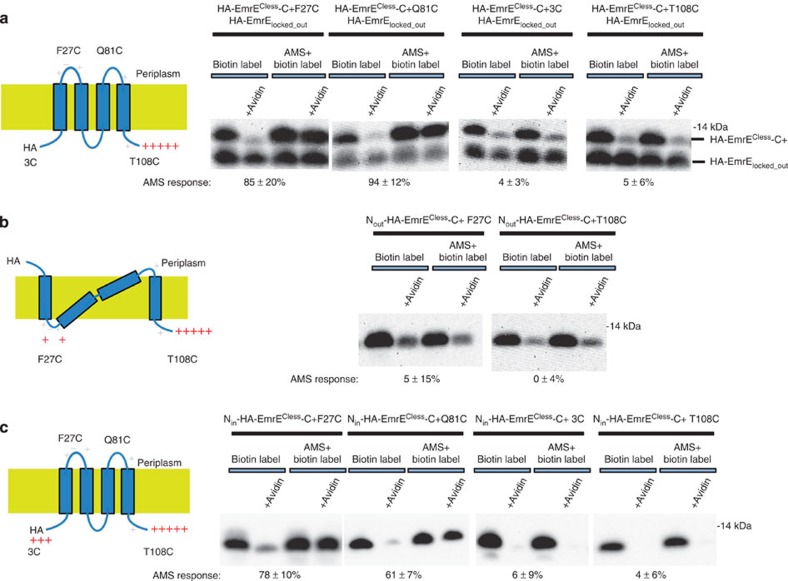
AMS reactivity tests for EmrE constructs. Proposed topologies are shown on the left and cysteine-reactivity data are shown on the right. Added positively charged residues are shown in red. Invariant positively charged residues present in the wild-type protein are shown in light grey. AMS response is the per cent change in biotinylation due to the reaction with AMS. (**a**) AMS response of different cysteines in the HA-EmrE^Cless^-C+ construct, when paired with HA-EmrE_locked_out_ to generate an antiparallel dimer. Unpaired HA-EmrE^Cless^-C+ subunits are rapidly degraded by FtsH. (**b**) Topology mapping for the construct N_out_-HA-EmrE^Cless^-C+. The frustrated N_out_/C_in_ topology shown is one of the many possible topologies. (**c**) Topology mapping for the construct N_in_-HA-EmrE^Cless^-C+. EmrE is visualized by western blotting for the HA epitope and the results shown are representative of assays performed in triplicate.

We considered the possibility that F27C is actually located fully in the periplasm in the HA-EmrE-C+ construct, but is only partially reactive to AMS because it is partially buried by protein or membrane (even though this does not occur in the EmrE-C+ construct missing the HA tag). To test whether F27C in HA-EmrE^Cless^-C+ would be fully blocked by AMS if the protein were in a pure N_in_/C_in_ topology, we expressed the single-cysteine variants of HA-EmrE^Cless^-C+ in a wild-type (FtsH^+^) strain in the presence of HA-EmrE_locked_out_. As illustrated above, when expressed in an FtsH^+^ strain, the N_in_/C_in_ topology remains resistant to proteolysis when expressed in the presence of HA-EmrE_locked_out_ because it can form stable dimers, but orphan, unpaired subunits, are rapidly degraded by FtsH. Thus, only the N_in_/C_in_ topology of HA-EmrE^Cless^-C+ will be present, allowing us to test the cysteine's reactivity in a pure N_in_/C_in_ subunit in the active dimer. As shown in Fig. 5a, when expressed with EmrE_locked_out_, F27C is fully blocked by AMS (85±20%, *n*=3), whereas T108C remained inaccessible to AMS (5±6%, *n*=3). Moreover, Q81C, in the third loop was fully blocked by AMS (94±12%, *n*=3), consistent with the N_in_/C_in_ topology when expressed with HA-EmrE_locked_out_. These results suggest that we could have seen complete blocking of the F27C position if HA-EmrE^Cless^-C+ adopted a pure N_in_/C_in_ topology.

It still remained possible that F27C in HA-EmrE^Cless^-C+ is more reactive to AMS in an active dimer compared with the orphan form in the absence of an N_out_/C_out_ subunit partner. To test this possibility, we attempted to generate pure normal N_in_/C_in_ and distorted N_out_/C_in_ topology orphan subunits by controlling the topology of the N-terminal helix. To favour an N_out_ orientation, we introduced a positive charge bias into loop 1 using the mutations G26R and T28R, to produce N_out_-HA-EmrE^Cless^-C+ (Fig. 5b). To favour an N_in_ orientation, we placed positive charges at the N terminus by changing the N-terminal sequence from MPNYIY to MRRRYIY, generating N_in_-HA-EmrE^Cless^-C+ (Fig. 5c). Because of the introduced charge biases, the N-terminal helix in these constructs should insert in unique orientations. Indeed for N_out_-HA-EmrE^Cless^-C+ construct, F27C and T108C no longer show any reaction to the AMS reagent (5±15% and 0±4%, respectively, *n*=3) indicating that F27C and T108C are cytoplasmic (Fig. 5b). Conversely, for N_in_-HA-EmrE^Cless^-C+, the F27C position is nearly fully blocked by AMS (78±10%, *n*=3) and 3C is completely unblocked (6±9%, *n*=3), consistent with an N_in_ topology (Fig. 5c). Q81C shows a less complete AMS response (61±7%, *n*=3), possibly due to an altered conformation in the monomeric subunit. Nevertheless, these results strongly suggest that the partial blockage observed for F27C in HA-EmrE^Cless^-C+ is not due to the changes in reactivity of F27C.

**Figure 6:**
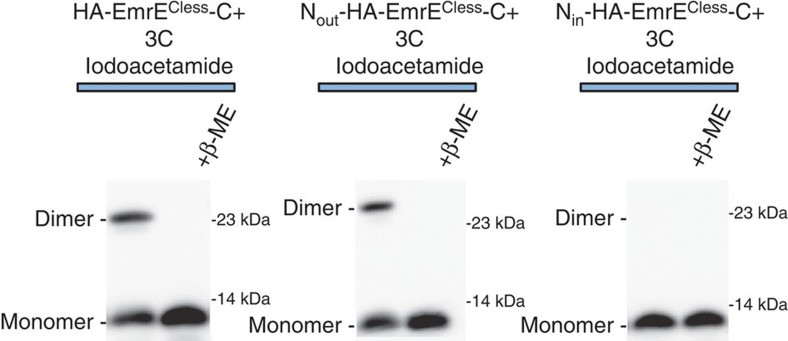
Disulfide formation at the N terminus. A 3C mutant in the context of the HA-EmrE^Cless^-C+ and N_out_-HA-EmrE^Cless^-C+ form dimers that are sensitive to the addition of 2-mercaptoethanol suggest that the 3C can form disulfide-linked dimers. No dimers were seen for the N_in_-HA-EmrE^Cless^-C+ construct. These results are consistent with a 3C location in the oxidizing periplasm for EmrE^Cless^-C+ and N_out_-HA-EmrE^Cless^-C+ constructs and a 3C location in the reducing cytoplasm for the N_in_-HA-EmrE^Cless^-C+ construct. Further disulfide formation was blocked by the addition of iodoacetamide prior to cell lysis. EmrE is visualized by western blotting for the HA epitope.

We were unable to use cysteine accessibility to map the location of 3C in the HA-EmrE^Cless^-C+ and N_out_-HA-EmrE^Cless^-C+ constructs because of disulfide bond formation of 3C. As shown in Fig. 6, a band at a dimer molecular weight is seen in constructs where 3C is expected to reside in the periplasm, but not in constructs where 3C is expected to reside in the cytoplasm. The dimer band is eliminated after reduction by β-mercaptoethanol (β-ME). Although disulfide formation complicates cysteine-accessibility analysis, formation of the disulfide bond is indicative of an oxidizing, periplasmic location. In N_in_-HA-EmrE^Cless^-C+, the 3C cysteine has robust reactivity with MPB and does not form disulfide bonds as would be expected for its cytoplasmic location (Fig. 6). Thus, the observation of disulfide formation is consistent with the predicted topologies.

### Partial reactivity is not due to topology flipping

**Figure 7:**
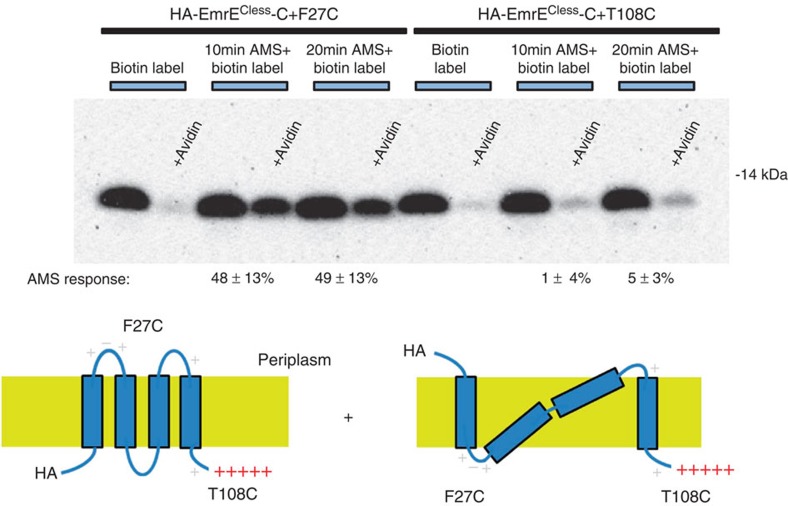
Time dependence of AMS reactivity. The extent of AMS blocking of HA-EmrE^Cless^-C+ F27C and T108C for 10 and 20 min is shown. Essentially no difference is observed, indicating that 10 min is sufficient to block all the available thiols in F27C and that the extent of reaction reflects an end point rather than a slowed rate of modification. The experimental conditions are the same as described in Fig. 4. EmrE is visualized by western blotting for the HA epitope.

We finally considered the possibility that the HA-tagged N terminus topology is not fixed in an N_in_ or N_out_ topology, but flips back and forth. If the N-terminal segment (including F27C) of HA-EmrE-C+ can move from the cytoplasm to the periplasm, then the partial reactivity could be due to its presence in the periplasm for only a fraction of the time. If so, the extent of F27C blocking should be time dependent. When we increased the AMS reaction time with HA-EmrE^Cless^-C+ F27C, however, we saw no increase in AMS blocking. When blocked for 10 or 20 min we observed AMS blocking of 48±13% (*n*=3) and 49±13% (*n*=3), respectively (Fig. 7). These results are consistent with a static topology of the inserted HA-EmrE-C+, that is, the protein is either N_in_/C_in_ or N_out_/C_in_ and does not flip after insertion.

## Discussion

Our results indicate that under normal circumstances EmrE inserts in two opposite topologies at roughly equal frequencies. In particular, with an HA tag at the N terminus that blocks subsequent topology flipping, we observe insertion in both an N_in_ and an N_out_ topology. As the initial insertion occurs in both topologies with roughly equal proportions, it suggests that the primary mechanism for EmrE dual-topology biogenesis is co-translational (Fig. 1a). While we cannot rule out global topology flipping in the wild-type protein as predicted by the post-translational mechanism, we only have direct evidence for more limited topology rearrangements when the protein is unnaturally inserted in a distorted topology. Indeed, to our knowledge our work is the first direct demonstration of topological malleability in EmrE since a static topology would produce the same results in the complementation assays used previously^12^ (see Fig. 1b). Moreover, we find that incorrectly inserted or unpaired subunits can be cleared rapidly by proteolysis so a mechanism for ensuring equal populations of both topologies by subsequent topology flipping is not necessary. When topological signals are manipulated by the placement of positive charges at the C terminus, causing initial insertion in a distorted N_out_/C_in_ topology, the topology can indeed rearrange to an N_in_/C_in_ topology as previously suggested by von Heijne and co-workers^12^. It is fascinating and remarkable that even partial topological rearrangements are possible, although they are likely driven by distorted topologies that may only rarely occur naturally. Thus, we suggest that major topology rearrangements are unlikely to play a major role in dual-topology generation with the normal EmrE topology signals.

## Methods

### Materials

AMS was purchased from Life Technologies (Grand Island, NY). MPB was purchased from Cayman Chemical (Ann Arbor, MI).

### Plasmids and constructs

The EmrE constructs were cloned into the pBAD/His A plasmid (Invitrogen) using the NcoI and XhoI cut sites, which removes the built-in tags. For the co-expression experiments, the EmrE_locked_out_ and EmrE_locked_in_ constructs were cloned into another pBAD vector, which contains a chloramphenicol-resistance gene and a ClodF13-derived CDF replicon. This vector was created from the pSEL1 vector described previously^18^. The CDF origin was removed from pCDFDuet-1 vector (Novagen) using the XbaI and NheI restriction sites and was ligated into the pSEL1 vector replacing the p15A origin using the same restriction sites. EmrE constructs were cloned into the vector using a NcoI and HindIII site. A second NcoI site in the chloramphenicol-resistant gene was removed by making a silent mutation of Thr172 from the ACC codon to the ACA codon using PCR quickchange mutagenesis.

HA-EmrE bears an N-terminal HA epitope (YPYDVPDYA) before the wild-type *Escherichia coli* (*E. coli*) EmrE sequence with an additional glycine downstream of the initial methionine. The amino acids KKKHHHHHH were added onto the C terminus for the constructs HA-EmrE-C+ after a linker (ENLYFQG). The EmrE-C+ construct has the same protein sequence as HA-EmrE-C+ except with a wild-type N terminus. A more detailed view of these constructs is given in Supplementary Fig. 5.

Surprisingly, HA-EmrE can be induced at full induction in the pBAD system without the cellular toxicity normally associated with the overexpression of EmrE^11^. Expression of the EmrE-C+ construct lacking the HA tag was toxic in our pBAD system. We speculate that the reduced toxicity of HA-EmrE may be due to a predicted RNA hairpin accidentally included in the HA tag that reduces expression (Supplementary Fig. 5). To use EmrE-C+ in our system, we therefore engineered a predicted RNA hairpin into EmrE-C+ by altering the DNA but not the protein sequence. The DNA mutations A-7T (in the pBad His A plasmid) and C6T prevent the toxic overexpression of EmrE-C+ in our pBAD system (Supplementary Fig. 5C,D). The N-terminal portion of the wild-type-EmrE construct contains the same DNA sequence as EmrE-C+.

All of the point mutations were introduced by quickchange PCR mutagenesis. The HA-EmrE_locked_out_ construct based on a previous work^11^ contains the N-terminal HA tag of HA-EmrE to prevent toxicity and the mutations T28R, L85R and R106A. The HA-EmrE_locked_in_ construct has the same HA tag to prevent toxicity and was also based on previous work^11^ but contains an additional mutation (M1K) to ensure complete topological locking in our system (M1K, R29G, R82S and S107K)^11^.

In all constructs used with cysteine-accessibility experiment (N_in_-HA-EmrE^Cless^-C+, N_out_-HA-EmrE^Cless^-C+, HA-EmrE^Cless^-C+ and EmrE^Cless^-C+), the native cysteine residues were changed to alanine (C39A, C41A and C95A). The added cysteines F27C, Q81C and T108C were then singly introduced into each of the cysteine null constructs. The mutation 3C adds the amino acids ‘CG' after the initial two N-terminal residues, MG, which are upstream of the HA epitope. To form the N_in_-HA-EmrE^Cless^-C+ construct, the HA-EmrE^Cless^-C+ construct had the mutations N2R, P3R and additional arginine inserted between the two residues. To form the N_out_-HA-EmrE^Cless^-C+ construct, the HA-EmrE^Cless^-C+ construct had the mutations G26R and T28R incorporated into sequence.

### Ethidium bromide-resistance assay

*E. coli* BL21Pro (Addgene/ClonTech) cells with the relevant plasmids were grown to saturation (∼10 h at 37 °C) and then serially diluted 10-fold six times. Five microlitres of each dilution was spotted onto plates with 0.2% arabinose, 34 μg ml^−1^ chloramphenicol, 100 μg ml^−1^ ampicillin, and the indicated concentration of EtBr. The plated cells were grown at 37 °C for 18 h.

### Cysteine-accessibility topology assay

AR3291 (FtsH null) cells^16^ containing the desired EmrE construct were grown to ∼0.8 OD_600_ and then induced with 0.2% arabinose at 30 °C for 2 h. Cells from 50 ml of cell culture were collected by centrifugation and suspended in 500 μl of 50 mM phosphate buffer with 17 mM NaCl at pH 8.0. Two-hundred microlitres of resuspended cells were incubated in a final concentration of 2 mM AMS for 10 min, rotating in the dark at room temperature. The cells were then washed twice with 50 mM Tris-Cl at pH 7.5. The final cell pellet was resuspended to a final volume of ∼200 μl. Samples not reacted with AMS underwent the same procedure without the AMS reagent. The cells were further incubated with 4 mM MPB in 4% dimethylsulphoxide, 0.5% toluene and 50 mM Tris-Cl at pH 7.5 for 1 h while rotating in the dark at room temperature. The cells were then lysed by sonication. Cell debris was removed by centrifugation at 16,000*g* for 10 min and then membranes were isolated by ultracentrifugation of the supernatant in a Beckman Coulter Airfuge at 160,000*g* for 1 h at room temperature. Membranes were resuspended in 20 mM Tris-Cl at pH 7.5. Total protein was determined by the DC Protein Assay (Bio-Rad). For each lane, 24 μg of protein was mixed with 4 × SDS loading buffer. Either 25 μg of avidin (Sigma BioUltra) in 20 mM Tris-Cl at pH 7.5 or 20 mM Tris-Cl at pH 7.5 was added to the samples and the samples were run on a 12% NuPAGE Bis-Tris (Life Technologies) gel using MES running buffer. The Accuruler Prestained Protein Ladder was used to estimate molecular weight. Electrophoresis was carried out at 40 V for 25 min and then 100 V for 135 min. The samples were then transferred to a (0.2 μm) PVDF membrane at 95 mA for 1 h using Towbin transfer buffer with 20% methanol.

For the HA antibody, the membrane was blocked with 5% non-fat milk in TBS-T (20 mM Tris-Cl at pH 7.5, 150 mM NaCl, 0.1% Tween 20) followed by an hour incubation in a 1:1,000 dilution of 1 mg ml^−1^ monoclonal HA antibody (Sigma #H3663) in TBS-T with 0.5% non-fat milk. The blot was then washed four times in 20 mM Tris-Cl at pH 7.5, 500 mM NaCl, 0.2% Triton X-100 and 0.05% Tween 20 buffer (TBS-Tween/Triton). The blot was incubated for 1 h in a 1:3,000 dilution of anti-mouse IgG peroxidase conjugate (Sigma #A4416) in TBS-T with 0.5% non-fat milk and then washed six times for 10 min in TBS-Tween/Triton buffer. Western blots for the histidine tag were performed as described above except that the membrane was blocked and blotted with the supplied blocking buffer and the pentahis horseradish peroxidase conjugate antibody (Qiagen #34460) as described in the QIAexpress detection and assay handbook (1:1,000 dilution).

The blots were visualized on a FluorChem FC2 (Alpha Innotech) CCD imager using the Amersham ECL Prime detection reagent (GE Healthcare) according to the recommended protocol. The intensity of the bands was quantified using ImageJ. A general background subtraction obtained from a dark area of the blot was applied to each band. The amount of biotinylation was determined by the decrease in band intensity between the lanes with and without avidin. The per cent change in biotinylation between the reaction with MPB only and the reaction with both AMS and MPB (labelled AMS response) was used to determine the location of each cysteine. An uncropped blot image is shown in Supplementary Fig. 6.

### CCL EmrE degradation assay

BL21Pro *E. coli* cells co-expressing HA-EmrE^Cless^-C+ with either N_out_-HA-EmrE^Cless^-C+ or N_in_-HA-EmrE^Cless^-C+ were grown to ∼0.8 OD_600_ and induced with 0.2% arabinose for 1 h at 30 °C. The cells were washed twice with LB media and then switched into LB media containing 350 μg ml^-1^ erythromycin and 0.2% glucose to stop further protein synthesis. Samples were taken at time zero (after the media switch) and at the other indicated time points.

### FtsH-null expression test

AR3291 cells (FtsH null) or AR3289 (control cells) obtained from the Ogura Lab^16^ were grown to ∼0.8 OD_600_ and then induced with 0.2% arabinose for 2 h at 30 °C. The cells were collected by centrifugation and lysed by sonication. The membranes were isolated and the samples were visualized by western blot for the HA epitope as described previously.

### Disulfide bond determination

AR3291 (FtsH null) cells containing the desired EmrE construct were grown to ∼0.8 OD_600_ and then induced with 0.2% arabinose at 30 °C for 2 h. Cells from 50 ml of cell culture were collected by centrifugation and suspended in 500 μl of 50 mM Tris-Cl buffer at pH 7.5. Two-hundred microlitres of resuspended cells were incubated in a final concentration of 5 mM ioacetamide for 15 min, rotating in the dark at room temperature. The cells were then washed twice with 50 mM Tris-Cl at pH 7.5 and lysed by sonication. Cell debris was removed by centrifugation at 16,000*g* for 10 min and then membranes isolated by ultracentrifugation of the supernatant in a Beckman Coulter Airfuge at 160,000*g* for 1 h at room temperature. Isolated membranes were resuspended in 20 mM Tris-Cl at pH 7.5. Twenty-four micrograms of the samples were then mixed in SDS loading buffer with 2-mercaptoethanol (β-ME) at 170 mM and without. The protein was visualized by western blot using the HA epitope as described earlier.

## Additional information

**How to cite this article:** Woodall, N. B. *et al.* Dual-topology insertion of a dual-topology membrane protein. *Nat. Commun.* 6:8099 doi: 10.1038/ncomms9099 (2015).

## Supplementary Material

Supplementary Information Supplementary Figures 1-6
