# Supplementary material for: Dual-topology insertion of a dual-topology membrane protein
Source: Nat Commun. 2015 Aug 26;6:8099. doi: 10.1038/ncomms9099 (PMC4560821; doi:10.1038/ncomms9099)
Supplement: Supplementary Information — Supplementary Figures 1-6 [file ncomms9099-s1.pdf]

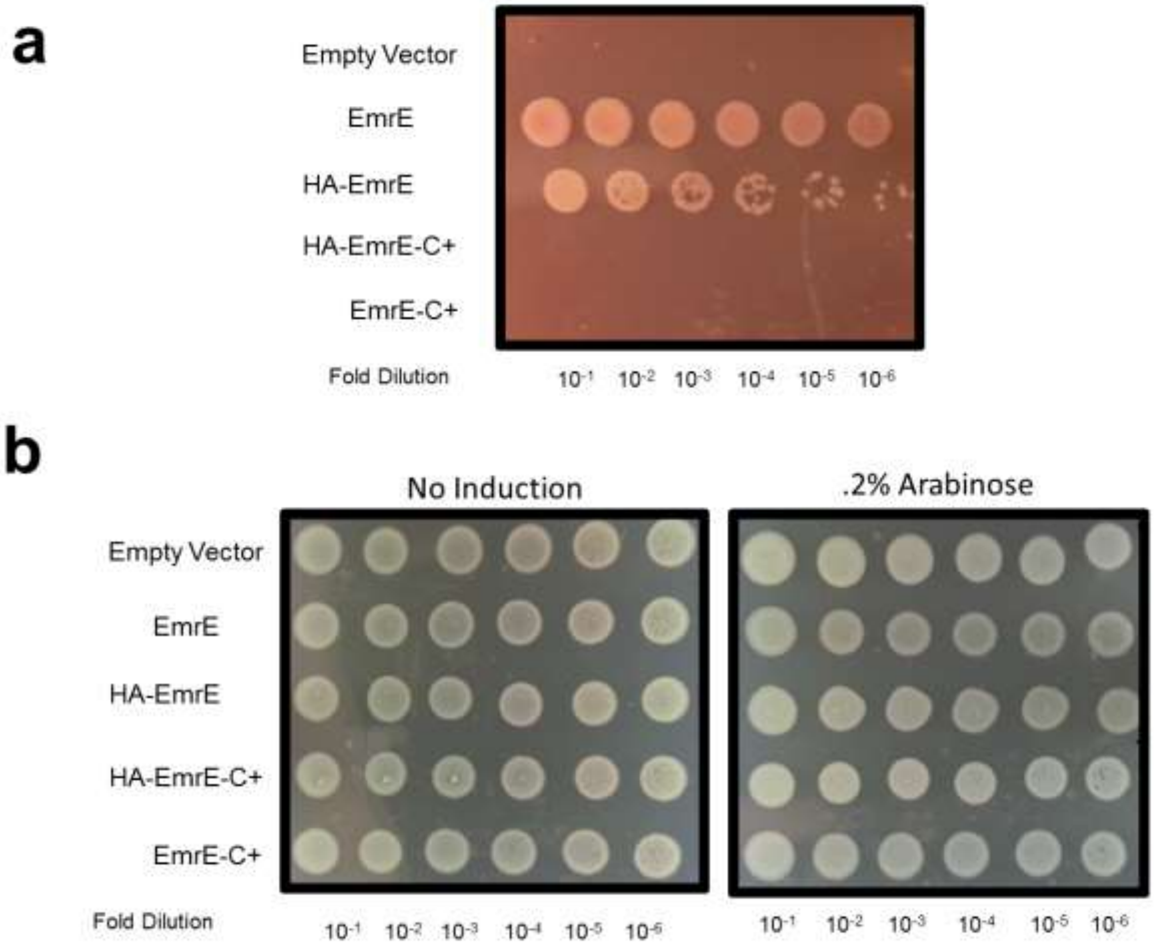

Supplementary Figure 1. Ethidium Bromide Resistance of HA-EmrE. (A) The growth of 10-fold dilutions of stationary phase BL21Pro cell culture expressing the indicated constructs spotted on agar plates in the presence of 220µg/mL ethidium bromide. **(B)** The growth of 10-fold dilutions of stationary phase BL21Pro cell culture with the indicated constructs on an arabinose inducible plasmid spotted on agar plates. The induction of the constructs at .2% arabinose is not toxic.

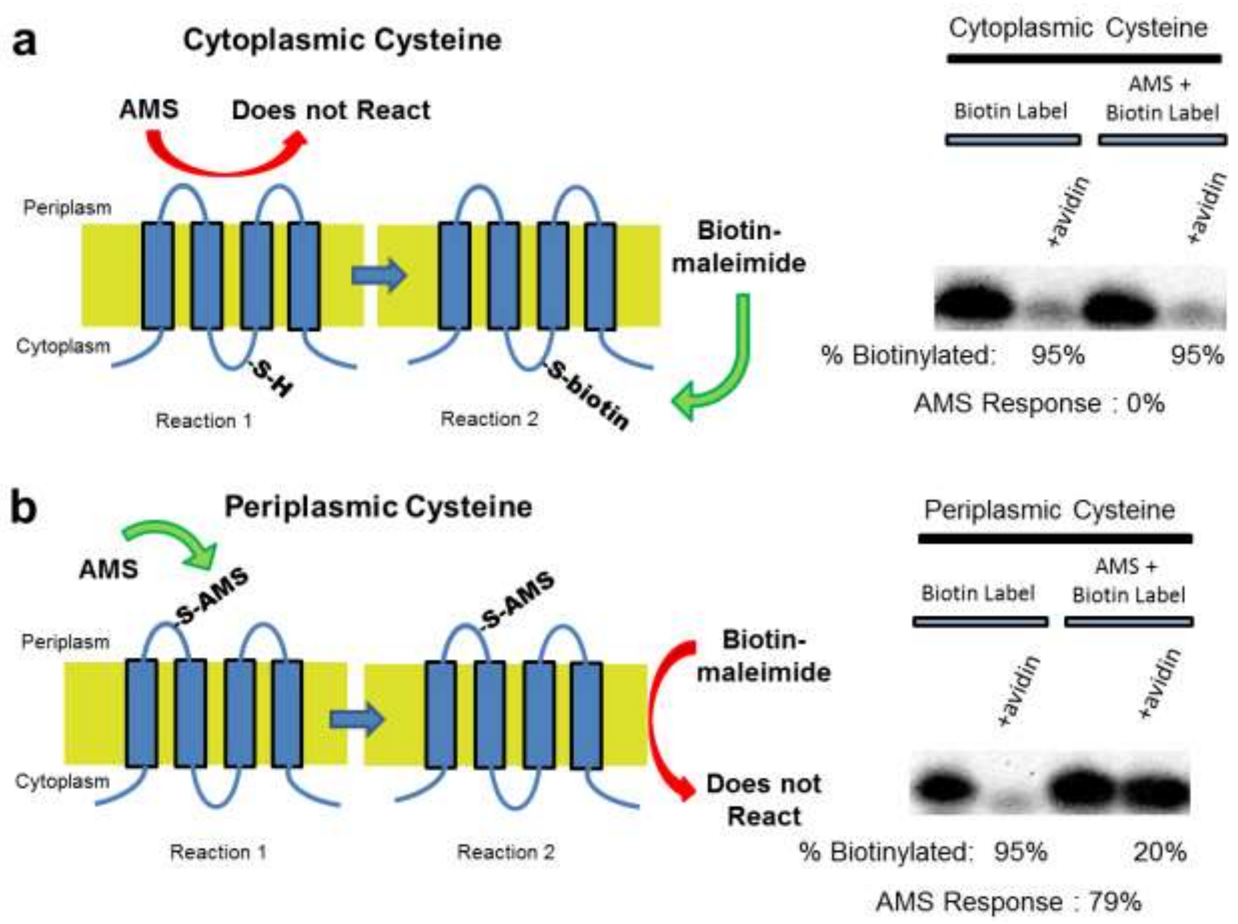

Supplementary Figure 2. Cysteine- Accessibility Method. Illustration of our specific application of the cysteine accessibility topology determination method. We determine whether a single cysteine in EmrE resides in either the cytoplasm or periplasm by its reactivity with a membrane-impermeable maleimide, AMS. Since the reaction of AMS is not easily detectable, we assess the amount of AMS that is reacted with EmrE by its ability to block a second reaction with a biotin-maleimide (MPB). The amount of biotinylation is then quantified by the amount of protein that is gel-shifted upon the addition of avidin (detected as a loss of the gel band corresponding to free EmrE). For each trial, the level of biotinylation without the AMS pre-reaction is determined by a reaction with the biotin-maleimide alone. The percent difference in biotinylation between the biotin-maleimide reaction alone and the AMS/biotin maleimide series is termed “AMS response.” **(A)** In the case of a cytoplasmic cysteine, AMS cannot react with the cytoplasmic cysteine due to its inability to cross the membrane. The subsequently added biotin-maleimide can therefore react freely with the unblocked cysteine. Thus, for a cytoplasmic cysteine, we see near complete biotinylation with the biotin-maleimide alone (~95% of the band lost upon addition of avidin) and again near complete biotinylation when pre-reacted with AMS (~95% of the band lost upon addition of avidin). The low AMS response, 0%, is indicative of a cytoplasmic cysteine. **(B)** In the case of a periplasmic cysteine, AMS reacts with the lone cysteine. The biotin-maleimide added next cannot react with the protein as the cysteine has already reacted with AMS. The final result is near complete biotinylation with the biotin-maleimide alone (~95% of the band lost upon addition of avidin) and limited biotinylation when pre-reacted with AMS (~20% of the band lost upon addition of avidin). The high AMS response, 79% = 100*((95-20)/95), is indicative of a periplasmic cysteine.

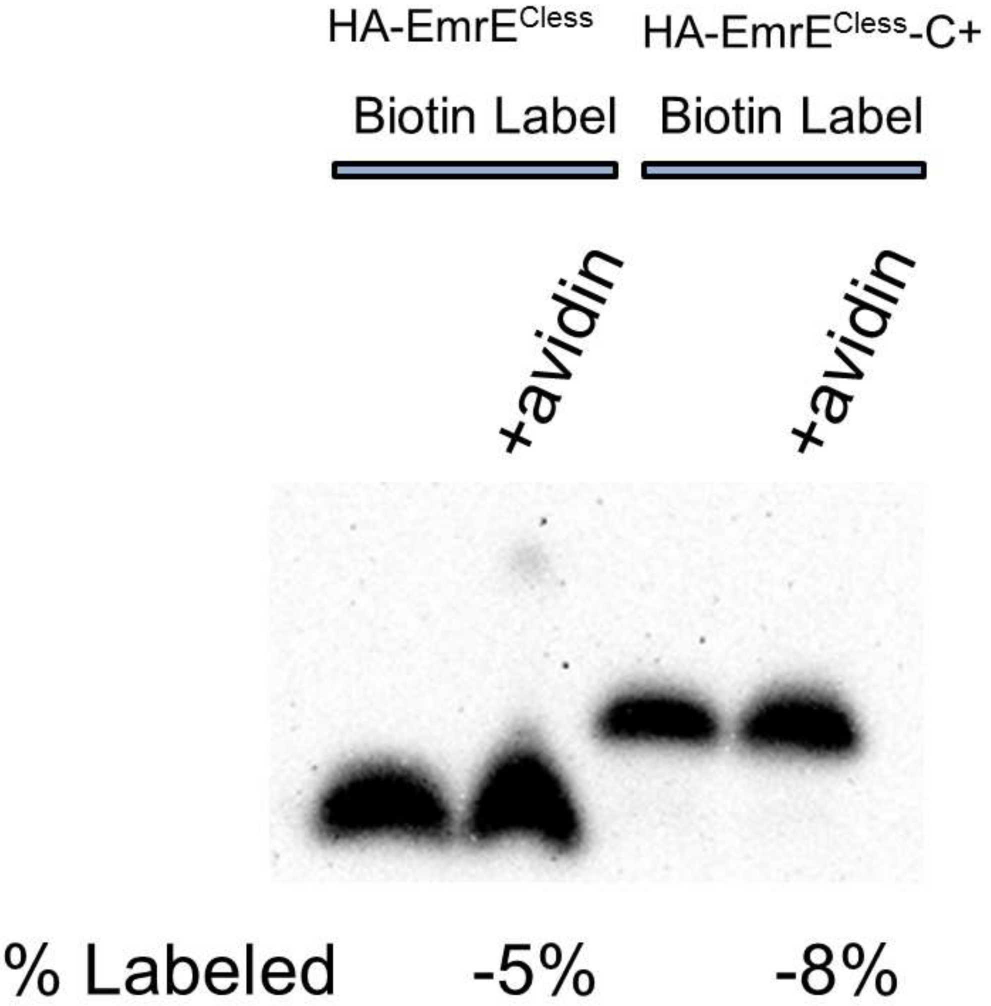

Supplementary Figure 3. Cysteine-Free Proteins are not Reactive. HA-EmrE^{Cless} and HA-EmrE^{Cless}-C+ are not biotinylated by MPB as shown by the absence of a gel shift with avidin. The proteins were visualized by western blot with a HA-antibody.

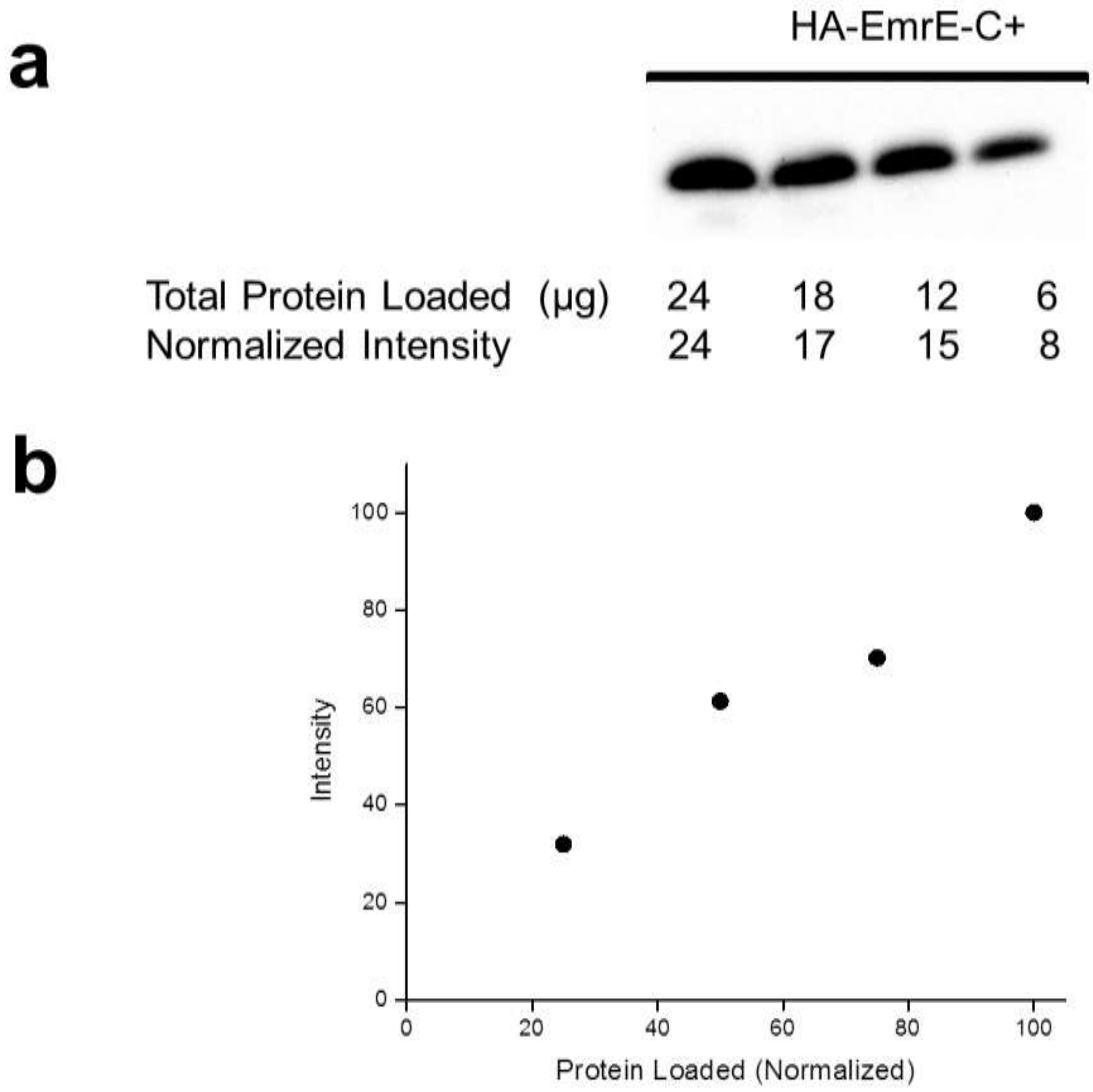

Supplementary Figure 4. Protein Amount is Linearly Related to Band Intensity. (A) A membrane preparation containing HA-EmrE-C+ was loaded into each well with the specified amount of total protein. The protein was visualized by western blot using the HA-epitope. **(B)** The band intensity from (A) plotted against the total protein loaded in the lane.

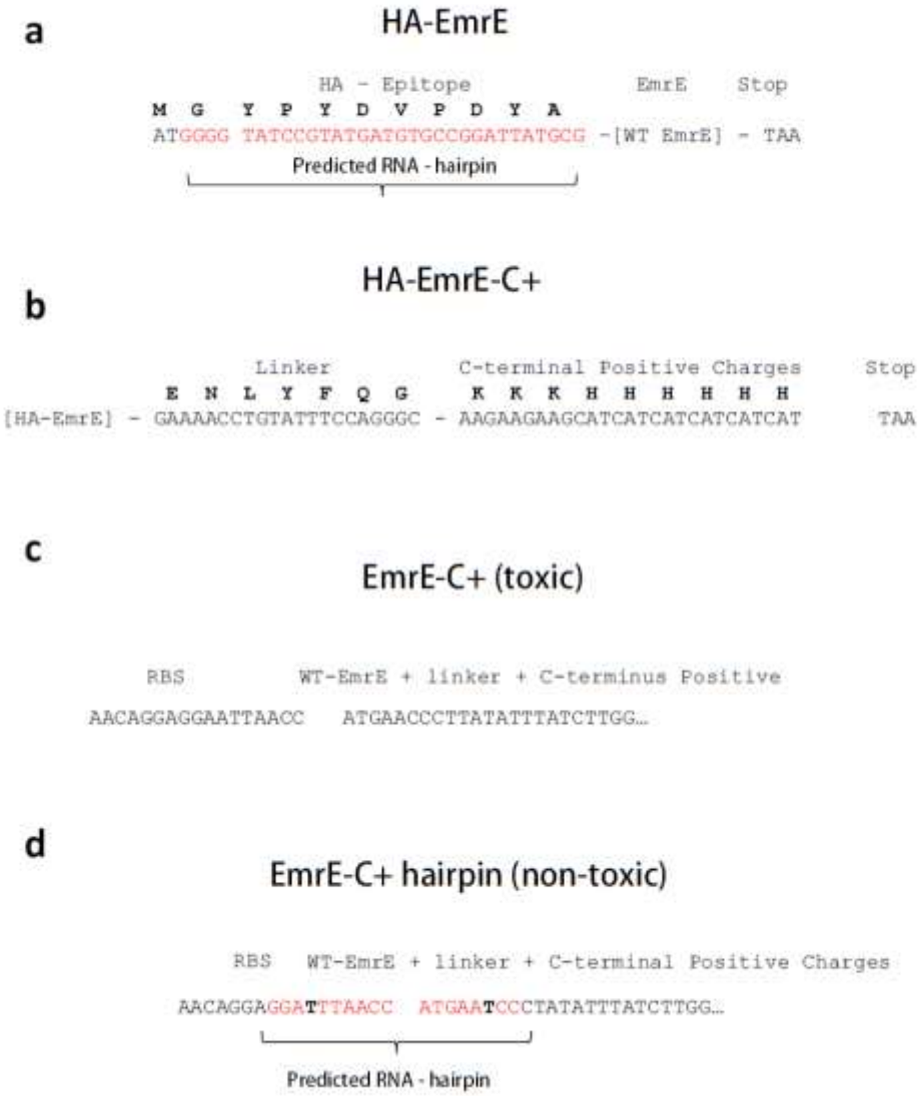

Supplementary Figure 5. DNA Sequences of EmrE Constructs. DNA sequence at the N- or C-termini for the indicated constructs. Regions that have a strong predicted RNA-hairpin are shown in a red font **(A)** HA-EmrE **(B)** HA-EmrE-C+ **(C)** EmrE-C+ (toxic) **(D)** EmrE-C+ hairpin (non-toxic) Mutations that do not alter the protein sequence to create a predicted RNA-hairpin are shown in a bold typeface.

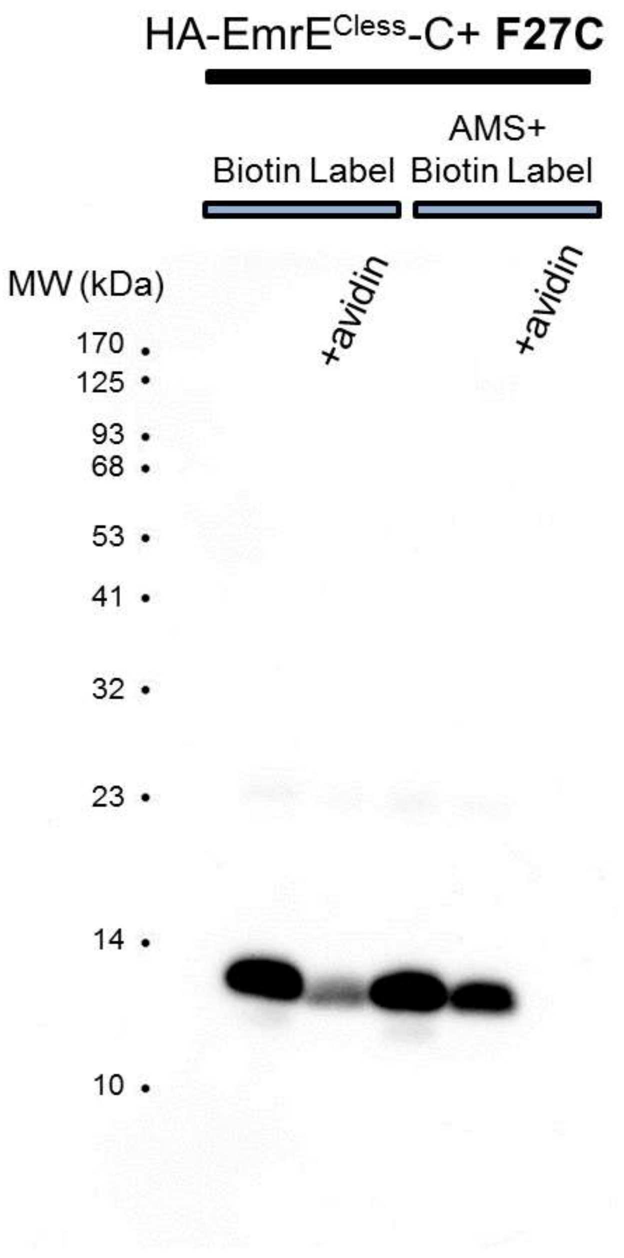

Supplementary Figure 6. Uncropped Blot of HA-EmrE^{Cless}-C+ F27C. Uncropped blot of cysteine accessibility assay for HA-EmrE^{Cless}-C+ F27C. The conditions are identical to the blot shown in Fig. 4B.
